# Supplementary material for: Menopause education of healthcare professionals: A scoping review protocol
Source: PLoS One. 2025 Nov 14;20(11):e0325012. doi: 10.1371/journal.pone.0325012 (PMC12617892; doi:10.1371/journal.pone.0325012)
Supplement: S2Appendix — (DOCX) [file pone.0325012.s002.docx]

S2 Appendix: Data extraction table 1

| **Authors/**  **Year** | **Country** | **Setting** | **Study Design** | **Population Type** | **Population Size** | **Study aims** | **Methodology** |
| --- | --- | --- | --- | --- | --- | --- | --- |
|  |  |  |  |  |  |  |  |
|  |  |  |  |  |  |  |  |
