# Supplementary material for: Menopause education of healthcare professionals: A scoping review protocol
Source: PLoS One. 2025 Nov 14;20(11):e0325012. doi: 10.1371/journal.pone.0325012 (PMC12617892; doi:10.1371/journal.pone.0325012)
Supplement: S3 Appendix — (DOCX) [file pone.0325012.s003.docx]

S3 Appendix: Data extraction table 2

| **Authors/**  **Year** | **Intervention** | **Facilitators** | **Participants/**  **Profession** | **Course delivery method** | **Course length** | **Course outline** |
| --- | --- | --- | --- | --- | --- | --- |
|  |  |  |  |  |  |  |
|  |  |  |  |  |  |  |
