# Supplementary material for: Menopause education of healthcare professionals: A scoping review protocol
Source: PLoS One. 2025 Nov 14;20(11):e0325012. doi: 10.1371/journal.pone.0325012 (PMC12617892; doi:10.1371/journal.pone.0325012)
Supplement: Attachment — Submitted filename: response to plos one final.docx [file pone.0325012.s005.docx]

**Menopause education of healthcare professionals: A scoping review protocol**

Thank you for considering our manuscript for publication in PLOS ONE. 

We thank the Editor and the Reviewers for their evaluation of our manuscript. Overall, we believe revisions have led to an improved paper. Please find below the detailed responses for each Reviewer. Additionally, we have attached the revised paper with all modifications highlighted.

| **Editor** | | |
| --- | --- | --- |
| **Comment** | **Our response** | **Where the change appears** |
| 1. When submitting your revision, we need you to address these additional requirements.    Please ensure that your manuscript meets PLOS ONE's style requirements, including those for file naming. The PLOS ONE style templates can be found at  <https://journals.plos.org/plosone/s/file?id=wjVg/PLOSOne_formatting_sample_main_body.pdf> and  <https://journals.plos.org/plosone/s/file?id=ba62/PLOSOne_formatting_sample_title_authors_affiliations.pdf> | We confirm our manuscript meets PLOS ONE’s style requirements |  |
| 2. Thank you for stating the following financial disclosure:  The authors would like to acknowledge that C. Keye is in receipt of a PhD scholarship from the School of Pharmacy, University College Cork, Cork, Ireland.    Please state what role the funders took in the study.  If the funders had no role, please state: "The funders had no role in study design, data collection and analysis, decision to publish, or preparation of the manuscript."  If this statement is not correct you must amend it as needed.  Please include this amended Role of Funder statement in your cover letter; we will change the online submission form on your behalf. | Thank you for your comment. We amended this statement as follows:  The authors would like to acknowledge that C. Keye is in receipt of a PhD scholarship from the School of Pharmacy, University College Cork, Cork, Ireland. The funder had no role in study design, data collection and analysis, decision to publish, or preparation of the manuscript. |  |
| 3. When completing the data availability statement of the submission form, you indicated that you will make your data available on acceptance. We strongly recommend all authors decide on a data sharing plan before acceptance, as the process can be lengthy and hold up publication timelines. Please note that, though access restrictions are acceptable now, your entire data will need to be made freely accessible if your manuscript is accepted for publication. This policy applies to all data except where public deposition would breach compliance with the protocol approved by your research ethics board. If you are unable to adhere to our open data policy, please kindly revise your statement to explain your reasoning and we will seek the editor's input on an exemption. Please be assured that, once you have provided your new statement, the assessment of your exemption will not hold up the peer review process. | Data Availability: No datasets were generated or analysed during the current study. This article describes a protocol for a scoping review. The full results and any associated datasets will be made available in a subsequent publication upon completion of the scoping review. |  |
| 4. If the reviewer comments include a recommendation to cite specific previously published works, please review and evaluate these publications to determine whether they are relevant and should be cited. There is no requirement to cite these works unless the editor has indicated otherwise. | N/A |  |
| 5. Please review your reference list to ensure that it is complete and correct. If you have cited papers that have been retracted, please include the rationale for doing so in the manuscript text, or remove these references and replace them with relevant current references. Any changes to the reference list should be mentioned in the rebuttal letter that accompanies your revised manuscript. If you need to cite a retracted article, indicate the article’s retracted status in the References list and also include a citation and full reference for the retraction notice. | N/A |  |

| **Reviewer 1** | | |
| --- | --- | --- |
| **Comment** | **Our response** | **Where the change appears** |
| In the search strategy, it is advisable to also search citing articles of known studies, so I would include this in the strategy. Also, with Open Grey not having been updated recently (archived in 2021), instead of searching this repository, I would suggest instead searching the websites of known organizations involved in menopause and/or medical and health care professional education. For example, FIGO and its members or the Menopause Society. You can likely use the peer-reviewed literature to find references to useful groups. | Thank you for that information. We have now removed open grey from the paper. Professional organisations, such as the FIGO, British Menopause Society, International Menopause Society and European Menopause and Andropause Society, have their own journals, all of which are indexed in PubMed and relevant papers have been included. We included an explanation of this. We also explained that we will conduct a hand search of the included papers. | Line 152-156 |
| Will the searches be included in the appendix of the final paper or archived somewhere else? This should be clear. | The full searches will be included in the full scoping review, which is being undertaken at the moment. We have now included this in the protocol. Thanks for your suggestion. | Line 219-221 |
|  |  |  |
|  |  |  |

| **Reviewer 2** | | |
| --- | --- | --- |
| **Comment** | **Our response** | **Where the change appears** |
| In the search phase, consider including the following websites to help identify pertinent unpublished sources.  EQUATOR Network (<https://www.equator-network.org/>); Centre for Evidence-Based Medicine (<https://www.cebm.net/>); Centre for Reviews and Dissemination (<https://www.york.ac.uk/crd/>); Critical Appraisal Skills Programme (<https://casp-uk.net/>); McMaster's Centre for Evidence-Based Medicine (<https://hslmcmaster.libguides.com/c.php?g=306765&p=2044668>); Evidence Synthesis International (<https://evidencesynthesis.org/>); JBI ([https://JBI.global/](https://jbi.global/)); Campbell Collaboration (<https://campbellcollaboration.org/>). | Thank you for your suggestion. We have now included these in the search. | Line 156-165 |
|  |  |  |
