# Supplementary material for: Menopause education of healthcare professionals: A scoping review protocol
Source: PLoS One. 2025 Nov 14;20(11):e0325012. doi: 10.1371/journal.pone.0325012 (PMC12617892; doi:10.1371/journal.pone.0325012)
Supplement: Attachment — Submitted filename: Reviewer 1.docx [file pone.0325012.s006.docx]

Reviewer 1

Thank you for the opportunity to review this protocol. It is well written according to the JBI standard and needs very few minor revisions.

In the search strategy, it is advisable to also search citing articles of known studies, so I would include this in the strategy. Also, with Open Grey not having been updated recently (archived in 2021), instead of searching this repository, I would suggest instead searching the websites of known organizations invovled in menopause and/or medical and health care professional education. For example, FIGO and its members or the Menopause Society. You can likely use the peer-reviewed literature to find references to useful groups.

Will the searches be included in the appendix of the final paper or archived somewhere else? This should be clear.

Reviewer 2

In the search phase, consider including the following websites to help identify pertinent unpublished sources.

EQUATOR Network (https://www.equator-network.org/);

Centre for Evidence-Based Medicine (https://www.cebm.net/);

Centre for Reviews and Dissemination (https://www.york.ac.uk/crd/);

Critical Appraisal Skills Programme (https://casp-uk.net/);

McMaster's Centre for Evidence-Based Medicine (https://hslmcmaster.libguides.com/c.php?g=306765&p=2044668);

Evidence Synthesis International (https://evidencesynthesis.org/);

JBI (https://JBI.global/);

Campbell Collaboration (https://campbellcollaboration.org/).
